# Longitudinal associations between fast food outlet count and inflammatory markers in the US-based nurses’ health study II between 1998 and 2011

**DOI:** 10.1016/j.numecd.2025.104476

**Published:** 2025-11-27

**Authors:** Noreen Z. Siddiqui, Joline W.J. Beulens, Jaime E. Hart, Jochem O. Klompmaker, Joreintje D. Mackenbach, Maria G.M. Pinho, Eric B. Rimm, Peter James

**Affiliations:** a Amsterdam UMC, Location Vrije Universiteit Amsterdam, Epidemiology and Data Science, Amsterdam, the Netherlands; b Amsterdam Public Health, Health Behaviors and Chronic Diseases, Amsterdam, the Netherlands; c University Medical Centre Utrecht, the Netherlands; d Department of Environmental Health, Harvard T.H. Chan School of Public Health, Boston, MA, USA; e Channing Division of Network Medicine, Department of Medicine, Brigham and Women's Hospital and Harvard Medical School, Boston, MA, USA; f Upstream Team, Amsterdam, the Netherlands; g Copernicus Institute of Sustainable Development, Utrecht University, Utrecht, the Netherlands; h Department of Nutrition, Harvard TH Chan School of Public Health, Boston, MA, 02115, USA; i Department of Epidemiology, Harvard T.H. Chan School of Public Health, Boston, MA, USA; j Department of Population Medicine, Harvard Medical School and Harvard Pilgrim Health Care Institute, Boston, MA, USA; k Department of Public Health Sciences, University of California Davis School of Medicine, Davis, CA, USA

**Keywords:** Food environment, Fast food outlets, Inflammation, Cardiometabolic health outcomes

## Abstract

**Background and aims:**

Inflammation is an established cardiovascular disease risk factor, but its role in the link between food environments and cardiovascular risk remains unexplored.

We aimed to study longitudinal associations between residential fast food outlets (FFOs) and inflammatory markers in US females from the Nurses’ Health Study II with stored blood and residential addresses.

**Methods and results:**

We counted FFOs within 1500-m buffers around each address in 1998 and 2010. In samples collected at two time points (1999, 2011), we measured C-reactive protein (CRP, N = 1350), Interleukin-6 (IL-6, N = 809), and adiponectin (N = 836). We performed multivariable linear regression with repeated measures to study changes in FFOs and inflammatory markers and multivariable linear regression analyses to study FFOs count in 1998 and changes in inflammatory markers between 1999 and 2011. Models were adjusted for age, race/ethnicity, partners’ education, smoking, neighborhood socioeconomic status (nSES), and population density. We explored effect modification by nSES and population density. No associations were observed in linear mixed models (e.g., CRP (β: 0.00, 95 %CI: 0.01,0.01) or in linear models including changes in inflammatory outcomes (e.g., CRP (β:0.00, 95 %CI: 0.01, 0.02). We also observed no effect modification for nSES or population density.

**Conclusion:**

In conclusion, we found no evidence for longitudinal associations between FFOs count and inflammatory markers in this study.

## 1. Introduction

Chronic diseases such as cardiovascular diseases, obesity, cancer, and diabetes are prevalent in the United States (US), with significant socioeconomic and healthcare burdens [1,2]. Diet is an important driver of these chronic diseases and may, in part, be influenced by the food environment, which has changed considerably over time [3,4]. This change is marked by a notable increase of community-level access to fast food outlets (FFOs), that are characterized by selling high-energy and ultra-processed foods [3-5].

Exposure to FFOs in the residential area may be linked to cardiovascular disease (CVD) outcomes by shaping dietary preferences. Previous studies found that higher residential exposure to FFOs was associated with a higher risk of cardiovascular disease outcomes [6-8]. Although research findings are inconsistent [9,10], some studies have shown that fast food outlet (FFO) exposure around the residence is associated with unhealthier dietary patterns, including higher consumption of meals rich in salt, saturated fat, sugar sweetened beverages, lower fruit and vegetable intake, and even larger portion sizes as a cause of the change in the food environment in some countries [11-14]. Altogether, this may potentially increase the risk of chronic diseases [9, 10].

Chronic low-level inflammation may serve as an important pathway linking FFOs, diet and health, as it is a foundational underpinning for many cardiovascular conditions. If left untreated or not reduced through lifestyle modifications, inflammation may lead to early onset of chronic diseases [15]. More specifically, C-reactive protein (CRP) levels and interleukin-6 (IL-6) are common inflammatory markers that have been consistently associated with cardiovascular diseases in prior research [16-18]. In contrast, adiponectin is considered a protective factor that helps reduce inflammation in the body and may lower the risk of chronic diseases [19,20].

However, no prior studies have examined how FFOs count might be linked to inflammation biomarkers. Our aim was therefore to study whether a higher count of FFOs around the home is associated with elevated levels of inflammatory markers in longitudinal analyses in US females.

## 2. Methods

### 2.1. Study design and study population

This study was embedded in the Nurses' Health Study II (NHSII), a large prospective population-based cohort study initiated in 1989 including 116,429 female registered nurses. In 1989, participants were between 25 and 42 years of age and resided in California, Connecticut, Indiana, Iowa, Kentucky, Massachusetts, Michigan, Missouri, New York, North Carolina, Ohio, Pennsylvania, South Carolina, and Texas. However, throughout follow-up, participants have moved and taken up residence across every state in the US. All residential addresses throughout the study for each participant were geocoded to derive latitude and longitude coordinates [21,22]. Detailed information regarding the NHSII has previously been published [23]. The study protocol was approved by the Institutional Review Boards of the Brigham and Women's Hospital and the Harvard T.H. Chan School of Public Health.

**Fig. 1.**
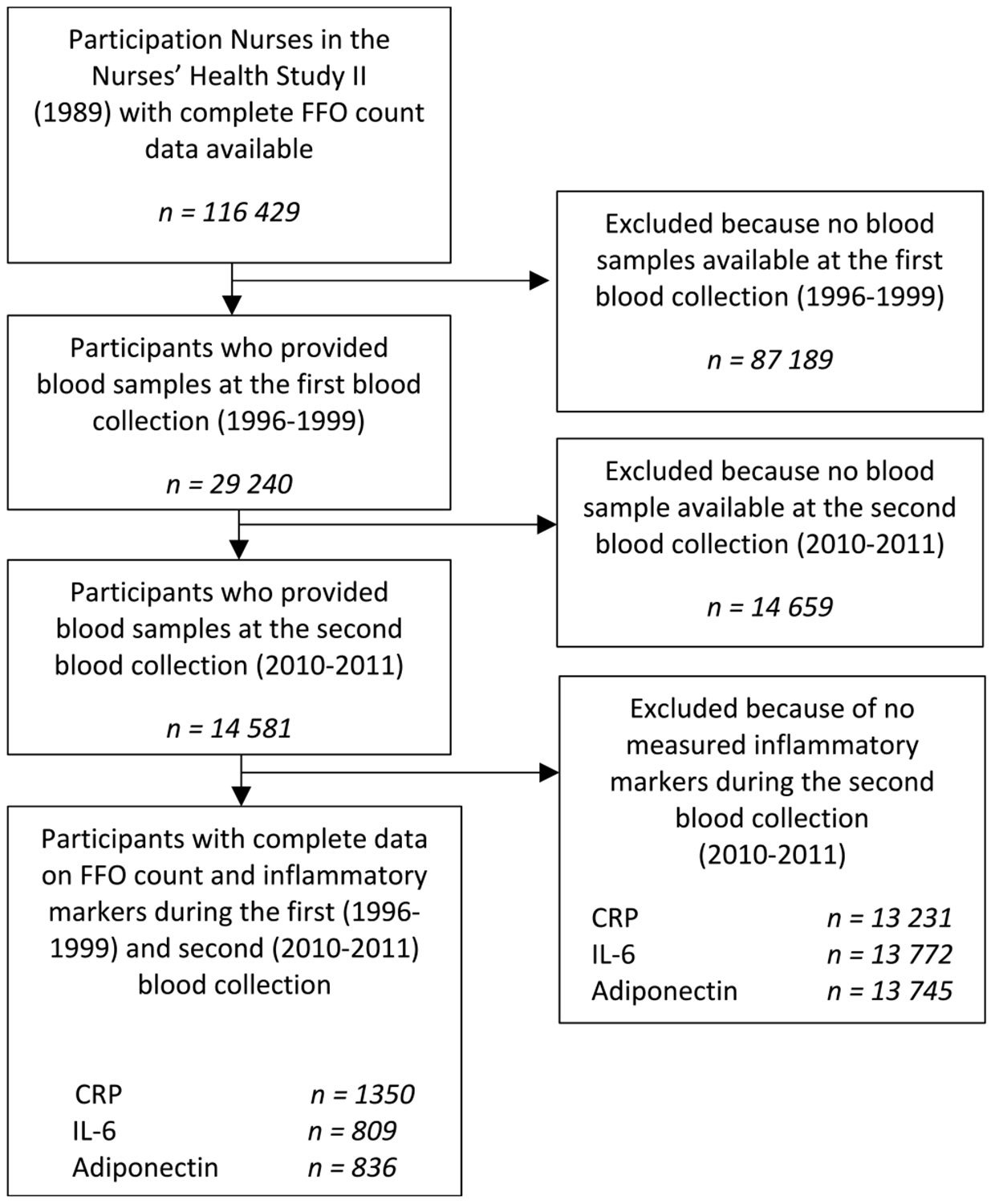
Flowchart study population.

Blood samples were collected in 1996–1999 (n = 29,611) and 2010–2011 (n = 17,275) among participants that were free of diagnosed cancer, diabetes, heart disease, or stroke and agreed to provide a blood sample [24]. The second blood collection only included participants who provided blood samples during the first blood collection [23]. A phlebotomy kit (including sodium heparin blood tubes, needles, and a tourniquet) was sent to the participants and returned by mail [25]. Subsequently, the samples were centrifuged to separate plasma from blood samples and were frozen until samples were analyzed [25]. In our analyses we included all participants who provided blood samples that had been analyzed for one of our inflammatory markers of interest and had address information available (Figure 1).

### 2.2. Exposure

#### 2.2.1. Food environment

Business data in 1998 and 2010 were obtained from Data Axle (Infogroup) Historical Business data [26]. This data includes geocoded information of all businesses in the US and is updated every two years. Each file consists of a “snapshot” of Data Axle's data as of the last day of each year. To address potential misclassification of FFO establishments, all establishments with ≥20 locations across the US were classified into the following categories: “full-service restaurant”, “fast food restaurant”, “supermarket or grocery store”, and “convenience store” [26]. Establishments with <20 locations were added to these categories based on the North American Industry Classification System 6-digit (NAICS6) codes, which are used by the Federal Statistical agencies for classifying business establishments [27]. Limited-service restaurants, as well as snack and non-alcoholic beverage bars identified by NAICS6 codes, were included in the “fast food restaurant” category [26]. By using Google Earth Engine and R, we developed 100 m rasters covering the contiguous US for 1998 and 2010 where each pixel represented the count of FFOs in a 1500 m buffer [26]. We then linked these data to the temporally appropriate participant's residential address.

For all our statistical models we linked FFO counts around the 1998 address to blood measurements collected in 1996–1999 and we linked FFO counts in 2010 to blood measurements collected in 2011.

### 2.3. Outcome

#### 2.3.1. Inflammatory markers

In the Nurses’ Health Study II cohort, plasma samples were analyzed for the following biomarkers: C-reactive protein (CRP), Interleukin 6 (IL-6), and adiponectin. These biomarkers were measured for a series of nested case-control studies within the NHSII, where samples were all non-cases at time of blood draw but cases were confirmed subsequently and matched and analyzed with their controls, as described in details elsewhere [23,28]. In brief, these studies were conducted to study various outcomes, such as type 2 diabetes, CVD, and several types of cancer. CRP was measured by using high sensitivity immunoturbidimetric assay on a BN II (Dade Behring, Newark, DE) [25]. Participants were excluded when concentrations were more than 10 mg/L, as this may indicate infection or medication use [24,29]. IL-6 was assessed by a quantitative sandwich enzyme immunoassay technique (Quantikine HS Immunoassay kit; R&D Systems, Minneapolis) and adiponectin concentrations were measured by using a competitive radioimmunoassay (Linco Research) [24].

### 2.4. Covariates

Information on individual-level covariates was available from biennial follow-up questionnaires. Since this cohort only includes female nurses who all have an undergraduate and potentially graduate educational background, participants were asked about their partners’ education as a proxy for individual socioeconomic status (SES). This was categorized into < high school, high school graduate, and >high school. Race/ethnicity was dichotomized into white or non-white. Smoking habits were categorized into current (including dose of cigarettes/day), past, or never. We accounted for neighborhood SES (nSES) by using Census tract-level variables that were obtained for the temporally closest Census from the Neighborhood Change Database (NCDB), which provides US Census data from 1999 to 2017, with normalized boundaries over time [30]. We calculated the nSES score by z-standardizing and summing the values of the following nine variables: median household income; median home value; neighborhood percentage of college degree holders; neighborhood percentage of non-Hispanic White; non-Hispanic Black; foreign-born residents; percentage of families receiving interest or dividends; percentage of occupied housing units, and percentage of unemployment. Higher values reflected higher nSES [30]. Population density was classified into ≥1000 persons/mile^2^ and <1000 person-s/mile^2^ Census tracts.

### 2.5. Statistical analyses

We evaluated associations using linear mixed models with FFO counts from 1998 and 2010, along with inflammatory markers from 1999 and 2011. Additionally, we used linear regression models to study the associations between FFO counts in 1998 and the changes in inflammatory markers between 1999 and 2011. Each biomarker (CRP, IL-6, adiponectin) was modelled separately. Given their differences in biological responses, we assessed linearity with splines and interpreted directions of associations relative to prespecified expectations (positive for CRP and IL-6 and inverse for adiponectin). In our crude model, we included counts of FFOs and inflammatory markers separately. In our fully adjusted model we adjusted for age, ethnicity, partners’ education level, smoking, nSES, and population density. We explored the linearity assumption using natural cubic splines, which indicated that associations were linear.

As results may differ within urban areas and between levels of nSES, we included population density and nSES as interaction terms in our second model and report the effect estimates of the interaction terms if we observed a statistically significant interaction (P-value for interaction <0.05).

In sensitivity analyses, we repeated our analyses in the subset of participants who did not change residences between 1998 and 2010. This was done to explore whether any associations between changes in FFO counts and inflammatory markers are explained by the actual change in the FFO environment (longitudinal associations in those who did not move) or through a change in exposure due to moving behaviors (longitudinal associations in movers). Since biomarkers were measured for a series of nested case-control studies, we also conducted a sensitivity analysis specifically excluding cases from each case-control study. Missing data of categorical covariates, smoking (0.1 %) and partner's education (13.6 %) were accounted for with a missing indicator [31], by creating a new binary variable that indicates whether the original variable had a missing value. Missing data for the continuous variable, nSES (0.3 %), was accounted for by carrying last available value from follow-up questionnaires forward or, if not available, by using the median value. All analyses were analyzed with SAS version 9.4.

## 3. Results

Our study population included participants who had at least one address and one biomarker measured in 1999 and 2011 (CRP, N = 1350; IL-6, N = 809; adiponectin, N = 836). Participants in the subsets with available IL-6 and adiponectin measurements had a median age of 46.8 (interquartile range (IQR): 43.5–49.8) years. This was similar to the subset with complete CRP data, in which participants had a median age of 46.5 (IQR:43.1–49.8) years. More than half of the participants in all subsets had partners with more than a high school education (CRP: 57.9 %, IL-6: 56.5 %, adiponectin: 56.6 %) (Table 1). Furthermore, our results showed a lower count of FFOs at follow-up (1.0 (0–7.0)) than at baseline (2.0 (0.0–7.0)). Inflammatory markers were higher at follow-up, with higher levels observed for CRP (1.2 mg/dl (0.6–2.6)) and adiponectin (7919 ng/ml (5709–10454))

We observed no associations between FFO count in 1998 and 2010 and the levels of CRP, IL-6, and adiponectin in 1999 and 2011 in linear mixed models (e.g., CRP (β: 0.00, 95 %CI: 0.01,0.01) (Table 2). In Table 3, results from models including FFO count in 1998 and the change in CRP, IL-6, and adiponectin between 1999 and 2011 showed no association between baseline FFO count and change in inflammatory markers. For example, for each count of FFO at baseline, CRP levels changed by β:0.00 mg/dl (95 %CI: 0.01,0.02) over the subsequent 12 years. Within our study sample we also found no evidence for effect modification between FFO count and neighborhood socioeconomic status, or population density (P-for-interaction >0.05).

In our sensitivity analyses, where we restricted our analyses to the control group (CRP, N = 429; adiponectin, N = 232; IL-6, N = 226), results remained similar across all models (Supplemental Tables 1-2). Similarly, in our sensitivity analyses including non-movers only (CRP, N = 990; adiponectin, N = 623; IL-6, N = 608), we observed similar effect estimates for associations between FFO count and CRP, IL-6, and adiponectin. (Supplemental Tables 3-4).

## 4. Discussion

We hypothesized that a higher FFO count around the home would be associated with easier access to and greater intake of food from FFO and therefore with elevated levels of inflammatory markers in longitudinal analyses. However, our study did not provide evidence to support these hypotheses. Similar results were found when we repeated our analyses in a subset of controls or participants who did not move.

A previous clinical study including postmenopausal women in New Zealand reported that a higher fast food pattern was associated with higher CRP levels [32]. This study also reported that dietary patterns rich in fruits and vegetables were associated with lower CRP levels [32]. Research, including from women in the Nurses’ Health Studies, further shows that a Western dietary pattern, including higher intake of red and processed meats, sweets, desserts, French fries, is linked to elevated inflammatory markers [33]. Additionally, studies have indicated that greater consumption of ultra-processed foods is associated with elevated levels of inflammatory markers [34-36]. Other studies showed that greater exposure to FFOs was associated with greater fast food consumption, unhealthy dietary patterns, a higher BMI, a higher odds of obesity and an increased risk of cardiovascular diseases [34,37-42]. However, these findings were not always consistent across studies [43-45], with some studies even showing associations in the opposite direction [46-49]. Although stronger and more consistent associations between the food environment and health outcomes are often found in the US compared to studies conducted in other countries [37-42]. For example, in a previous study it was found that higher exposure to fast food outlets was associated with a poorer dietary intake compared to lower exposure to fast food outlets in the US [50,51]. We did not find evidence for this association for inflammatory markers in our population of US female nurses when we assessed FFO exposure based solely on proximity to home address.

There are several potential explanations for why we did not find associations between FFOs and inflammatory markers. First, our data was limited to counts of FFOs measured surrounding an individuals’ residential address, excluding exposure to FFOs around other locations such as work. Previous studies suggested that taking into account both home and work locations might better capture the food environment that individuals are exposed to [46,52,53]. Second, we used data based on FFOs within a buffer around residential addresses. We did not measure if, nor how often, individuals visit that FFO. While incorporating information on frequency of visits to FFOs would be valuable, such data address a slightly different research question, namely actual utilization. Third, we focused on FFOs solely, as existing literature on cardiovascular diseases has predominantly indicated associations with FFO [40]. However, unhealthy food items can also be purchased at other food outlets such as supermarkets, convenience stores, work access to FFO or even as gifts in the work environment from thankful patients [54].

In addition, our results showed that exposure to the count of FFOs was lower at follow-up, which contradicted our initial hypothesis and previous literature suggesting that FFOs increase over time [3,55]. While this may still be the case, it might apply only to areas outside the residential areas we examined in our study. Previous research observed a higher number of FFOs in urban areas and areas with lower SES [56, 57]. Thus, the observed reduction in exposure may reflect a shift in participant residence rather than a true environmental decline. In addition, as participants aged, they may have moved to quieter or more rural environments, thereby reducing their exposure to FFOs. We also observed higher levels of CRP and adiponectin at follow-up. However, we could not attribute this increase in inflammatory markers to FFOs, as no associations were found in our analyses. Higher levels of inflammatory markers, such as CRP, may be explained by other factors that we did not explore in this study, such as changes in dietary patterns [58]. Finally, the small sample size may not be powered to detect an association or there may simply be no association between FFOs and inflammatory markers in this population. In addition, our analyses may have been underpowered to detect interaction effects, particularly for IL-6 and adiponectin due to their smaller sample sizes, as such analyses may require larger samplesd.

### 4.1. Strengths and limitations

Strengths of this study include our study design, which allowed us to study longitudinal associations among participants from a well-characterized population-based cohort. We derived detailed information regarding inflammatory markers and several potential confounding factors, such as smoking. Additionally, we assessed FFO within buffers around residential addresses, whereas other US studies have used Census-tract-based FFO exposure. Another strength of this study is that we had address data that was updated every two years and that we were able to conduct sensitivity analyses in a subset of non-movers. Nevertheless, it is important to acknowledge the limitations of this study.

Firstly, we only had information about residential FFO exposure. Participants likely visited locations outside of their residential area, for example when they were on their way to work or other locations, also known as their activity space [59]. Additionally, our data did not provide information regarding choice of FFOs, purchases, or frequency of visiting these FFOs. This could lead to a misclassification of FFO exposure, particularly considering that supermarkets may offer both healthy and unhealthy food options, including ultra-processed foods that may increase inflammation [60]. Although collecting such data would be valuable, this was beyond the scope of our aim. Finally, we may not have enough variability in FFO count or may have used too small of a buffer zone for which a participant would typically walk or drive to access a FFO.

Moreover, some blood samples were collected between 1996 and 1999, while the FFO exposure was measured in 1998, potentially leading to a temporal mismatch wherein the outcomes have been assessed before the exposure for certain blood samples. Nonetheless, we made the assumption that there was a minimal change in the exposure over these years.

### 4.2. Implications and recommendations for future studies

Based on our study and the results of previous studies investigating geographical exposure to FFOs (e.g., density, proximity), which similarly yielded inconsistent results, there could be a possibility that FFO count may not drive dietary behaviors and therefore does not affect inflammation. There may also be other aspects of the food environment that should be explored, such as the totality of access to food retailers, the affordability of food or the marketing for fast food on top of physical availability. It would be recommended for future studies to incorporate more detailed measures of exposure to the food environment, for example by incorporating questions about individuals’ usage of nearby food outlets, frequency of visits, and typical orders/purchases. This addition, alongside studying the counts of FFOs, would provide a comprehensive understanding of how individuals interact with their food environment.

In conclusion, while previous studies found an association between fast food intake and biomarkers of cardiovascular risk, we did not find evidence for longitudinal associations between the count of FFOs in the residential neighborhood and several inflammatory markers in a population of US female nurses. Future studies with larger samples and additional information on spatial exposures or food purchasing behaviors may help to further clarify these associations.

## Supplementary Material

1

## Appendix A. Supplementary data

Supplementary data to this article can be found online at https://doi.org/10.1016/j.numecd.2025.104476.

## Appendices.

**Table 1:** Characteristics of Nurses' Health Study II participants with fast food outlet and inflammatory data from 1999 to 2011

	C-reactive protein (CRP)	Adiponectin	Interleukin 6 (IL-6)
	n = 1350	n = 836	n = 809
	% or median (25–75 percentile)	% or median (25–75 percentile)	% or median (25–75 percentile)
*Exposure assessment*			
Count of fast food outlets within a 1500 m buffer in 1999	2.0 (0–8.0)	2.0 (0.0–7.0)	2.0 (0.0–7.0)
Count of fast food outlets within a 1500 m buffer in 2011	1.0 (0–7.0)	1.0 (0.0–7.0)	1.0 (0.0–7.0)
*Outcome assessment*			
CRP in 1999 (mg/dl)	1.1 (0.5–2.7)	–	–
CRP in 2011 (mg/dl)	1.2 (0.6–2.6)	–	–
Adiponectin in 1999 (ng/ml)	–	6853 (5147–9398)	–
Adiponectin in 2011 (ng/ml)	–	7919 (5709–10454)	–
IL-6 in 1999 (pg/ml)	–	–	0.8 (0.6–1.2)
IL-6 in 2011 (pg/ml)	–	–	0.8 (0.6–1.2)
*Covariates*			
Education partner, more than high school graduate (%)	57.9	56.6	56.5
Smoking, current smoker (%)	4.3	3.6	3.3
Neighborhood socioeconomic status (z-score)	−0.6 (−2.6–1.9)	−0.8 (−2.8–1.8)	−0.7 (−2.7–1.9)
Population density (number per km^2^, ≥1000 km^2^ (%))	52.7	51.8	52.2
*Demographic characteristics*			
Age (years)	46.5 (43.1–49.8)	46.8 (43.5–49.8)	46.8 (43.5–49.8)
Race, white (%)	98.2	97.6	97.7

Table 1 shows the descriptive characteristics of nurses who participated in the Nurses' Health Study. Variables are presented in percentages (%) or presented as median (25–75 percentile).

**Table 2:** Results of linear mixed models of fast food outlet counts (1998–2010) and inflammatory outcomes (1999–2011)

	CRP (mg/dl)	Adiponectin (ng/ml)	IL-6 (pg/ml)
	n = 1350	N = 836	N = 809
*Crude model β (95 % CI)*			
Per 1 count of FFO	−0.00 (−0.01, 0.01)	−5.28 (−20.92, 10.36)	0.00 (−0.00, 0.00)
*Fully adjusted model β (95 % CI)*			
Per 1 count of FFO	−0.00 (−0.01, 0.01)	−12.25 (−28.47, 3.98)	0.00 (−0.00, 0.01)

Regression coefficients (β) and 95 % confidence intervals (CIs) from multivariate linear regression models, representing associations between the count of fast food outlets (FFO) within a 1500 m buffer and inflammatory markers.

Crude model: Crude associations between fast food outlets and inflammatory markers. Fully adjusted model: adjusted for age, ethnicity, partner's education, smoking, neighborhood socioeconomic status, and population density.

**Table 3:** Results of multivariable models of fast food outlet counts (1998) and changes in inflammation (1999–2011)

	CRP (mg/dl)	Adiponectin (ng/ml)	IL-6 (pg/ml)
	n = 1350	N = 836	N = 809
*Crude model β (95 % CI)*			
Per 1 count of FFO	0.00 (−0.01, 0.02)	−7.76 (−20.95, 5.44)	−0.00 (−0.00, 0.00)
*Fully adjusted model β (95 % CI)*			
Per 1 count of FFO	0.00 (−0.01, 0.02)	−9.22 (−23.23, 4.78)	−0.00 (−0.01, 0.00)

Regression coefficients (β) and 95 % confidence intervals (CIs) from multivariate linear regression models, representing associations between the count of fast food outlets within a 1500 m buffer and the change in inflammatory markers.

Crude model: Crude associations between fast food outlets and inflammatory markers. Fully adjusted model: adjusted for age, ethnicity, partner's education, smoking, neighborhood socioeconomic status, and population density.
