# Supplementary material for: Longitudinal associations between fast food outlet count and inflammatory markers in the US-based nurses’ health study II between 1998 and 2011
Source: Nutr Metab Cardiovasc Dis. Author manuscript; Available in PMC 2026 Jul 29. (PMC13415390; doi:10.1016/j.numecd.2025.104476)
Supplement: 1 [file NIHMS2187446-supplement-1.docx]

**Supplemental Table 1.** Results of linear mixed models in continuous values of the counts of FFOs in 1998 and 2010 within a 1500m buffer and inflammatory outcomes in 1999 and 2011, among the controls only

|  | CRP (mg/dl) n=429 | Adiponectin (ng/ml)  N=232 | IL-6 (pg/ml)  N=226 |
| --- | --- | --- | --- |
| *Crude model*  *β (95% CI)* | | |  |
| Per 1 count of FFO | -0.00 (-0.02, 0.01) | -7.44 (-48.02, 33.15) | -0.00 (-0.01, 0.01) |
| *Fully adjusted model*  *β (95% CI)* | | |  |
| Per 1 count of FFO | 0.00 (-0.01, 0.01) | -19.42 (-67.26, 28.42) | 0.00 (-0.01, 0.01) |
| Regression coefficients (β) and 95% confidence intervals (CIs) from multivariate linear regression models, representing associations between the count of fast food outlets (FFO) within a 1500m buffer and inflammatory markers.  Crude model: Crude associations between fast food outlets and inflammatory markers. Fully adjusted model: adjusted for age, ethnicity, partner’s education, smoking, neighborhood socioeconomic status, and population density. | | | |

**Supplemental Table 2.** Multivariable models in continuous values of the count of fast food exposure in 1998 within a 1500m buffer and change of inflammatory outcomes between the years 1999 and 2011, among the controls only

|  | CRP (mg/dl) n=429 | Adiponectin (ng/ml)  N=232 | IL-6 (pg/ml)  N=226 |
| --- | --- | --- | --- |
| *Crude model*  *β (95% CI)* | | |  |
| Per 1 count of FFO | -0.00 (-0.02, 0.01) | -16.14 (-50.39, 18.10) | 0.00 (-0.01, 0.01) |
| *Fully adjusted model*  *β (95% CI)* | | |  |
| Per 1 count of FFO | -0.00 (-0.02, 0.01) | -11.33 (-53.21, 30.55) | -0.00 (-0.01, 0.01) |
| Regression coefficients (β) and 95% confidence intervals (CIs) from multivariate linear regression models, representing associations between the count of fast food outlets within a 1500m buffer and the change in inflammatory markers.  Crude model: Crude associations between fast food outlets and inflammatory markers. Fully adjusted model: adjusted for age, ethnicity, partner’s education, smoking, neighborhood socioeconomic status, and population density. | | | |

**Supplemental Table 3.** Results of linear mixed models in continuous values of the counts of FFOs in 1998 and 2010 within a 1500m buffer and inflammatory outcomes in 1999 and 2011 in non-movers

|  | CRP (mg/dl) n=990 | Adiponectin (ng/ml)  N=623 | IL-6 (pg/ml)  N=608 |
| --- | --- | --- | --- |
| *Crude model*  *β (95% CI)* | | |  |
| Per 1 count of FFO | -0.01 (-0.02, 0.01) | -3.66 (-20.20, 12.88) | 0.00 (-0.00, 0.01) |
| *Fully adjusted model*  *β (95% CI)* | | |  |
| Per 1 count of FFO | -0.00 (-0.01, 0.01) | -10.25 (-27.29, 6.80) | 0.00 (-0.00, 0.01) |
| Regression coefficients (β) and 95% confidence intervals (CIs) from multivariate linear regression models, representing associations between the count of fast food outlets (FFO) within a 1500m buffer and inflammatory markers.  Crude model: Crude associations between fast food outlets and inflammatory markers. Fully adjusted model: adjusted for age, ethnicity, partner’s education, smoking, neighborhood socioeconomic status, and population density. | | | |

**Supplemental Table 4** Multivariable models in continuous values of the count of fast food exposure in 1998 within a 1500m buffer and change of inflammatory outcomes between the years 1999 and 2011 in non-movers

|  | CRP (mg/dl) n=990 | Adiponectin (ng/ml)  n=623 | IL-6 (pg/ml)  N=608 |
| --- | --- | --- | --- |
| *Crude model*  *β (95% CI)* | | |  |
| Per 1 count of FFO | 0.00 (-0.01, 0.02) | -5.45 (-20.20, 9.31) | -0.00 (-0.01, 0.00) |
| *Fully adjusted model*  *β (95% CI)* | | |  |
| Per 1 count of FFO | 0.00 (-0.01, 0.02) | -7.07 (-22.70, 8.56) | -0.00 (-0.01, 0.00) |
| Regression coefficients (β) and 95% confidence intervals (CIs) from multivariate linear regression models, representing associations between the count of fast food outlets (FFO) within a 1500m buffer and inflammatory markers.  Crude model: Crude associations between fast food outlets and inflammatory markers. Fully adjusted model: adjusted for age, ethnicity, partner’s education, smoking, neighborhood socioeconomic status, and population density. | | | |
